# Understanding Marginal Structural Models for Time-Varying Exposures: Pitfalls and Tips

**DOI:** 10.2188/jea.JE20200226

**Published:** 2020-09-05

**Authors:** Tomohiro Shinozaki, Etsuji Suzuki

**Affiliations:** 1 Department of Information and Computer Technology, Faculty of Engineering, Tokyo University of Science, Tokyo, Japan; 2 Department of Epidemiology, Graduate School of Medicine, Dentistry and Pharmaceutical Sciences, Okayama University, Okayama, Japan

**Keywords:** causal inference, g-formula, inverse probability weighting, marginal structural model, time-varying exposure

## Abstract

Epidemiologists are increasingly encountering complex longitudinal data, in which exposures and their confounders vary during follow-up. When a prior exposure affects the confounders of the subsequent exposures, estimating the effects of the time-varying exposures requires special statistical techniques, possibly with structural (ie, counterfactual) models for targeted effects, even if all confounders are accurately measured. Among the methods used to estimate such effects, which can be cast as a marginal structural model in a straightforward way, one popular approach is inverse probability weighting. Despite the seemingly intuitive theory and easy-to-implement software, misunderstandings (or “pitfalls”) remain. For example, one may mistakenly equate marginal structural models with inverse probability weighting, failing to distinguish a marginal structural model encoding the causal parameters of interest from a nuisance model for exposure probability, and thereby failing to separate the problems of variable selection and model specification for these distinct models. Assuming the causal parameters of interest are identified given the study design and measurements, we provide a step-by-step illustration of generalized computation of standardization (called the g-formula) and inverse probability weighting, as well as the specification of marginal structural models, particularly for time-varying exposures. We use a novel hypothetical example, which allows us access to typically hidden potential outcomes. This illustration provides steppingstones (or “tips”) to understand more concretely the estimation of the effects of complex time-varying exposures.

## BACKGROUND ON THE TOPIC

When we try to say something meaningful about a specific exposure–outcome causal relationship, counterfactual models are among the most popular and widely accepted approaches in the epidemiologic community.^1^^–^^6^ A counterfactual approach not only formalizes the language of cause and effect,^7^^–^^13^ but has also triggered the explosive development of novel analytic methods, including propensity scores (ie, the probability of exposure conditional on measured confounders)^14^^–^^19^ and regression model-based estimation methods (ie, multivariable-adjusted outcome modeling, possibly followed by averaging predicted risks under distinct exposure statuses),^20^^,^^21^ which have been evolved into doubly robust estimation.^22^^–^^28^ More importantly, a counterfactual approach has spurred extensive discussion on the assumptions for inferring causality from data and the conditions for specific statistical methods to work using, for example, causal diagrams.^2^^–^^4^^,^^6^^,^^29^^–^^35^ Yet, the most striking illustration brought about by the counterfactual approach may be that it can offer an elegant solution to the controversy surrounding the definition and estimability of the effects of exposures that vary over time. For example, initiated antiretroviral therapy (exposure) for acquired immunodeficiency syndrome may be intermitted after looking at the symptoms of pneumonia, which is a predictor of clinical outcomes (eg, death) but affected by the prior exposure, and thus considered as a part of the exposure’s effects. While no existent theory (at the time) in the statistics literature had offered clear guidance for adjusting or not adjusting for such intermediate variables to estimate the effect of time-varying exposures, new causal methodologies emerged in the 1980s. These include Robins’ unified approach, which is comprised of the generalized computational algorithm formula (abbreviated as g-formula) and estimation methods (ie, inverse probability weighting and g-estimation) of two classes of counterfactual, or structural, models.^36^^–^^42^

In 2000, *marginal structural models* were introduced as a tool to make the effects of such time-varying exposures easily estimable.^43^^–^^45^ Specifically, a marginal structural model is an equation to demonstrate prespecified assumptions on the causal effects to be estimated (ie, causal estimands). Thanks to the series of Robins and Hernán’s seminal works,^46^^–^^51^ as well as others’ tutorials on the topic with intuitive theory and easy-to-implement software,^52^^–^^59^ marginal structural models have been widely applied to longitudinal data. Herein, we illustrate the use of marginal structural models, parameters of which can be estimated in a comparative way using inverse probability weighting and the g-formula in certain situations, featuring hypothetical data with a time-varying exposure to point out common pitfalls as well as serve as a stepping stone to better understand the use of these methods.

## CONCEPTUAL PITFALLS

If readers feel confused with the following statements, they could be trapped by the pitfalls around the methodology considered in this paper:

1. Marginal structural models should be distinguished from inverse probability weighting.
2. A marginal structural model is an equation to show prespecified assumptions on causal estimands, while an exposure probability model for inverse probability weighting is an imposed restriction on observed distribution for estimation.
3. As a marginal structural model and exposure probability model (for inverse probability weighting) are used for different purposes, misspecification of these models would lead to biases in different ways.
4. Principles for variable selection for marginal structural models are distinct from that for exposure probability models, and thus model specification of them raises different challenges.
5. Inverse probability weighting shares identifiability assumptions with the g-formula and can be used to fit marginal structural models when the assumptions are met, although g-formula can be used to fit them only when the models are saturated.

Although some of these pitfalls have been appreciated previously,^57^ we aim to discuss them from a different perspective. Before entering these subtleties, it would be helpful to seize the rationale of the specialized causal methods elaborated for time-varying exposures with simple worked examples without relying on computerized packages. Unlike point-exposure settings,^1^^,^^6^^,^^60^^,^^61^ however, we rarely encounter such pedagogic examples of time-varying exposures, including counterfactual data that explicate causal estimands and underlying conditions. Although there are at least four excellent numerical examples appropriate for exercise, they rely on either the external causal knowledge (ie, causal diagrams without explicit estimands^51^^,^^59^ or “g-null” theorem implied by a causal diagram and observed data^6^) or “true” parameters for simulated data.^54^ In this paper, we provide a step-by-step illustration, or tips, using a novel, hypothetical numerical example dataset that includes potential outcomes, which directly incorporates minimal information to explicitly define causal estimands and conditions for their identification. One may consider a causal diagram would be helpful to understand the structure of the dataset. As noted later, however, causal diagrams typically include more causal assumptions than sufficient conditions to identify causal effects. That is why we do not start by drawing causal diagrams and use them only complimentarily in our illustration, despite the fact that they are indeed useful tools for explicating our assumptions in real data analysis.^35^

The following “tips” emanate from two introductory subsections regarding the effects of point exposures and time-varying exposures. Then, we step into the main contents to understand the unique role of and distinction between inverse probability weighting, marginal structural models, and regression/exposure probability models.

## TIPS TO UNDERSTAND WHAT, WHY, AND HOW OF MARGINAL STRUCTURAL MODELS

### Prerequisite: identification of point-exposure effects

As many epidemiologists become familiarized with a potential-outcome framework for a single time point, or a point-exposure setting, we just briefly review it here; readers unfamiliar with the basic concepts and notation may refer to Part 1 of *Causal Inference: What If*^6^ or concise introduction papers.^61^^,^^62^ Suppose that exposure *A_i_* (eg, antihypertensive drug), outcome *Y_i_* (eg, the occurrence of cardiovascular disease), and set of covariates *L_i_* (eg, current/prior health conditions, unhealthy behaviors, and social support) are observed for individual *i* = 1,…, *n*. Let *Y_i_^a^* denote the possibly unobserved, potential outcome that would be observed if, possibly counterfactually, exposure *A_i_* were set to level *a* = 0 (unexposed) or 1 (exposed) (hereafter, we may omit subscript *i* if no confusion will occur). Then, the average causal effect of exposure *A* on outcome *Y* may be defined as E[*Y*^1^] − E[*Y*^0^], which compares counterfactual expectations (or risks for a binary outcome) of *Y*^1^ and *Y*^0^ in the same population along the difference-scale.

Suppose a hypothetical cohort (Table 1) of 1,240 members whose E[*Y*^0^] = 660/1,240 = 0.532 and E[*Y*^1^] = 830/1,240 = 0.669, indicating moderate risk increase (causal risk difference of 13.7%) by exposure *A*. Note that in a counterfactual framework, because either *Y_i_*^0^ or *Y_i_*^1^ can be observed as *Y_i_* according to actual exposure status *A_i_*, we can observe neither E[*Y*^0^] nor E[*Y*^1^] directly in the data. Thus, we need the set of assumptions to identify the causal effect^1^^,^^6^^,^^14^^,^^61^: consistency (ie, if *A_i_* = *a* then *Y_i_* = *Y_i_^a^* for all *a*), positivity (ie, 0 < *P*(*A* = *a*|*L*) almost everywhere, for all *a*), and the following conditional exchangeability given covariates, say, *L*.

**Table 1.** Hypothetical cohort data with potential outcomes under point-exposure

Stratum		*N*	Potential outcome^a^				Observed outcome	
		
*L*	*A*		*Y*^0^ = 1	Risk	*Y*^1^ = 1	Risk	*Y* = 1	E[*Y*|*A*, *L*]
1	1	280	168	0.6	**210**	**0.75**	210	0.75
1	0	720	**432**	**0.6**	540	0.75	432	0.6
0	1	180	45	0.25	**60**	**0.333**	60	0.333
0	0	60	**15**	**0.25**	20	0.333	15	0.25

Total		1,240	660	0.532	830	0.669		

^a^Unobservable counterfactual distributions. Bold numbers are observed as *Y* = 1 (by consistency) in each stratum.

Table 1 also presents the observed distribution of (*L_i_*, *A_i_*, *Y_i_*) in accordance with potential outcome *Y_i_^a^* (*a* = 0, 1) under consistency. In Table 1, potential risk under *a* = 0 in the exposed E[*Y*^0^|*A* = 1] = 213/460 = 0.463 is not equal to that in the unexposed E[*Y*^0^|*A* = 0] = 447/780 = 0.573, and the same is true for potential risk under *a* = 1, E[*Y*^1^|*A*]. When *A* is associated with *Y^a^* as previously, marginal (unconditional) exchangeability is violated and the *A*–*Y* association (observed risk difference) is said to be *confounded*: E[*Y*|*A* = 1] − E[*Y*|*A* = 0] = 270/460 − 447/780 = 1.4%, indicating almost null association. Fortunately, within every strata of *L*, we can verify from Table 1 (“Risk” columns) that E[*Y^a^*|*A* = 0, *L* = *l*] = E[*Y^a^*|*A* = 1, *L* = *l*] (*a* = 0, 1 and *l* = 0, 1) and thus equal to E[*Y^a^*|*L* = *l*]. This condition is called “conditional exchangeability given *L*” and the sets of covariates that satisfy the condition are said to be *cofounders*.^6^^,^^36^ Under the condition, a weighted mean, or *standardized* risk,^4^^,^^52^ $\sum_{l}\text{E}$[*Y*|*A* = *a*, *L* = *l*]*P*(*L* = *l*) is equal to E[*Y^a^*]^6^; that is, causal effects are identifiable. In our data, standardized risks for *A* = 0 and *A* = 1 are

$$0.6(1,000/1,240)+0.25(240/1,240)=0.532=\text{E}[Y^{0}]\text{ and}$$

$$0.75(1,000/1,240)+0.333(240/1,240)=0.669=\text{E}[Y^{1}],$$

respectively.

The next subsection extends the definitions for and the conditions sufficient to identify causal effects for time-varying settings. To focus on the complexity of conditional exchangeability in time-varying settings, we suppose throughout this paper that consistency and positivity assumptions, as well as the time-varying versions of them,^51^^,^^63^^,^^64^ are met in our data.

### Definition and identification of effects of time-varying exposures

#### Targeted effects of time-varying exposure

If an exposure varies over time, the aforementioned definition of effects should be redefined. Consider a simple case with 2 time points. At time 1, baseline confounders *L*_1_*_i_* are measured and then exposure *A*_1_*_i_* is commenced; at time 2, confounder set *L*_2_*_i_* is measured and exposure is changed to *A*_2_*_i_*; finally, outcome *Y_i_* is measured. Thus, the observed data are (*L*_1_*_i_*, *A*_1_*_i_*, *L*_2_*_i_*, *A*_2_*_i_*, *Y_i_*), for *i* = 1,…, *n*. Note that *A*_1_ and *A*_2_ may represent the same exposure (eg, start/stop antihypertensive drugs) or different exposures introduced sequentially (eg, first-line and second-line chemotherapy for cancer patients). Likewise, *L*_1_ and *L*_2_ may consist of the same set of variables or (partly or entirely) distinct sets of variables.

For time-varying exposure, potential outcome can be defined by the combination of intervention on a joint exposure (*A*_1_*_i_*, *A*_2_*_i_*): let $Y_{i}^{a_{1},a_{2}}$ denote the potential outcome that would be observed if exposure *A*_1_*_i_* and *A*_2_*_i_* were set to level *a*_1_ and *a*_2_, respectively. We assume that exposure at each time takes on 0 (unexposed) or 1 (exposed), leading to 4 different potential outcomes—*Y_i_*^0,0^, *Y_i_*^0,1^, *Y_i_*^1,0^, and *Y_i_*^1,1^ for each individual *i*. The average causal effect of exposure on outcome may be defined as any contrast between counterfactual expectations E[$Y^{a_{1},a_{2}}$]; eg, E[*Y*^1,1^] − E[*Y*^0,0^]. We can also consider E[*Y*^1,0^] − E[*Y*^0,0^], which is referred to as the “controlled direct effect of *A*_1_ while *A*_2_ set at 0.”^65^^–^^67^

Note that joint exposure (*A*_1_, *A*_2_) can affect not only outcome *Y*, but also *L*_2_ (by *A*_1_), which is measured after exposure initiation. Under the implausible assumption of no effect of (the part of) exposure on (the part of) the following confounders, the effect of (*A*_1_, *A*_2_) can solely be seen as a multivalued exposure at a single time-point; as shown earlier, $\sum_{l_{1},l_{2}}\text{E}$[*Y*|*A*_1_ = *a*_1_, *A*_2_ = *a*_2_, *L*_1_ = *l*_1_, *L*_2_ = *l*_2_]*P*(*L*_1_ = *l*_1_, *L*_2_ = *l*_2_) is equal to E[$Y^{a_{1},a_{2}}$] if the corresponding exchangeability assumptions for point-exposure hold. In the following hypothetical data, however, there is no single set of confounders for joint effects of (*A*_1_, *A*_2_). Rather, *L*_1_ is a sufficient set of confounders for *A*_1_, and (*L*_1_, *A*_1_, *L*_2_) is a sufficient set of confounders for *A*_2_. This condition would enable us to identify E[$Y^{a_{1},a_{2}}$] but the usual standardization formula, $\sum_{l_{1},l_{2}}\text{E}$[*Y*|*A*_1_ = *a*_1_, *A*_2_ = *a*_2_, *L*_1_ = *l*_1_, *L*_2_ = *l*_2_]*P*(*L*_1_ = *l*_1_, *L*_2_ = *l*_2_) leads to biased estimates unless the aforementioned implausible assumption of no-effect of past exposures on time-varying confounders holds.^6^^,^^36^^,^^43^^,^^51^

#### A hypothetical cohort

For simplicity, consider a hypothetical cohort with empty *L*_1_. The situation would arise if *A*_1_ is randomized at baseline, but non-adherence occurs or another exposure is introduced during the follow-up, or if the cohort is restricted based on measured variables *L*_1_. In either case, the following illustration is unaffected by including the diverse values of *L*_1_, so let us ignore the adjustment for baseline confounders in our illustration.^6^^,^^51^^,^^54^ Table 2 provides the data distribution of (*A*_1_*_i_*, *L*_2_*_i_*, *A*_2_*_i_*, *Y_i_*) augmented by unobserved potential outcome $Y_{i}^{a_{1},a_{2}}$ (*a*_1_, *a*_2_ = 0, 1) in the hypothetical cohort. As in Table 1, observed outcome *Y_i_* coincides with $Y_{i}^{a_{1},a_{2}}$ such that (*A*_1_*_i_*, *A*_2_*_i_*) = (*a*_1_, *a*_2_) by consistency. We want to identify from observational data four expectations E[$Y^{a_{1},a_{2}}$] (“Total” row of “Risk” columns).

**Table 2.** Hypothetical cohort data with potential outcomes under time-varying exposure

Stratum			*N*	Potential outcome^a^								Observed outcome	
		
*A*_1_	*L*_2_	*A*_2_		*Y*^0,0^ = 1	Risk	*Y*^0,1^ = 1	Risk	*Y*^1,0^ = 1	Risk	*Y*^1,1^ = 1	Risk	*Y* = 1	E[*Y*|*A*_1_, *L*_2_, *A*_2_]
1	1	1	720	648	0.9	648	0.9	432	0.6	**576**	**0.8**	576	0.8
1	1	0	180	162	0.9	162	0.9	**108**	**0.6**	144	0.8	108	0.6
1	0	1	1,800	1,080	0.6	990	0.55	900	0.5	**720**	**0.4**	720	0.4
1	0	0	1,800	1,080	0.6	990	0.55	**900**	**0.5**	720	0.4	900	0.5
0	1	1	5,670	5,103	0.9	**4,536**	**0.8**	2,835	0.5	3,402	0.6	4,536	0.8
0	1	0	630	**567**	**0.9**	504	0.8	315	0.5	378	0.6	567	0.9
0	0	1	840	252	0.3	**294**	**0.35**	462	0.55	252	0.3	294	0.35
0	0	0	3,360	**1,008**	**0.3**	1,176	0.35	1,848	0.55	1,008	0.3	1,008	0.3

Total			15,000	9,900	0.66	9,300	0.62	7,800	0.52	7,200	0.48		

^a^Unobservable counterfactual distributions. Bold numbers are observed as *Y* = 1 (by consistency) in each stratum.

We note that neither unconditional nor conditional (given *L*_2_) exchangeability holds for joint exposure (*A*_1_, *A*_2_) in our data. For example, in the subgroups of (*A*_1_, *A*_2_) = (1, 1) and (0, 0), E[*Y*^0,0^|*A*_1_ = 1, *A*_2_ = 1] = 1,728/2,520 = 0.686 differs from E[*Y*^0,0^|*A*_1_ = 0, *A*_2_ = 0] = 1,575/3,990 = 0.395 (unconditional exchangeability fails). Likewise, E[*Y*^0,0^|*A*_1_ = 1, *L*_2_ = 0, *A*_2_ = 1] = 0.6 ≠ E[*Y*^0,0^|*A*_1_ = 0, *L*_2_ = 0, *A*_2_ = 0] = 0.3 (conditional exchangeability fails). Readers can see other potential outcomes $Y^{a_{1},a_{2}}$ also differ on average between distinct subgroups of (*A*_1_, *A*_2_). Next, let us see the bias in estimators ignoring or solely stratifying on *L*_2_ as a “baseline” confounder.

#### Naïve standardization vs the g-formula

Table 3 shows the observable part of Table 2 in a different layout, adding some candidate estimates from observed data. “*L*_2_-collapsed” estimates are risks in subgroups of joint exposure, E[*Y*|*A*_1_ = *a*_1_, *A*_2_ = *a*_2_] without considering *L*_2_. These are away from E[$Y^{a_{1},a_{2}}$] in Table 2 because of the lack of unconditional exchangeability. On the other hand, “naïve standardization” uses standardization formula in point-exposure settings: $\sum_{l_{2}}\text{E}$[*Y*|*A*_1_ = *a*_1_, *A*_2_ = *a*_2_, *L*_2_ = *l*_2_]*P*(*L*_2_ = *l*_2_), where *P*(*L*_2_ = 1) = 0.48 and *P*(*L*_2_ = 0) = 0.52. For example, standardized risk in (*A*_1_, *A*_2_) = (0, 0) can be obtained as

$$\begin{array}{rl} (567/630)0.48+(1,008/3,360)0.52 & =(0.9)0.48+(0.3)0.52\\ & =0.59. \end{array}$$

However, this estimate is (and other estimates are) again biased from E[*Y*^0,0^] = 0.66 (and other E[$Y^{a_{1},a_{2}}$] in Table 2) owing to the violation of conditional exchangeability given *L*_2_.

**Table 3.** Estimates of effects of time-varying exposure from hypothetical cohort data

	*A*_1_ = 0							*A*_1_ = 1							*p*(*L*_2_)
	
	*A*_2_ = 0			*A*_2_ = 1			*p*(*L*_2_|*A*_1_)	*A*_2_ = 0			*A*_2_ = 1			*p*(*L*_2_|*A*_1_)	
				
*L*_2_	*N*	*Y* = 1	Risk	*N*	*Y* = 1	Risk		*N*	*Y* = 1	Risk	*N*	*Y* = 1	Risk		
1	630	567	0.9	5,670	4,536	0.8	0.6	180	108	0.6	720	576	0.8	0.2	0.48
0	3,360	1,008	0.3	840	294	0.35	0.4	1,800	900	0.5	1,800	720	0.4	0.8	0.52

Estimates of E[$Y^{a_{1},a_{2}}$]^a^															
*L*_2_-collapsed^b^	3,990	1,575	0.39	6,510	4,830	0.74		1,980	1,008	0.51	2,520	1,296	0.51		
Naïve standardization^c^			0.59			0.57				0.55			0.59		
G-formula^d^			0.66			0.62				0.52			0.48		

^a^(*a*_1_, *a*_2_) corresponds to the value of (*A*_1_, *A*_2_).

^b^Calculate E[*Y*|*A*_1_, *A*_2_] using *N* and *Y* = 1 data in the subgroup defined by (*A*_1_, *A*_2_).

^c^Calculate $\sum_{l_{2}}\text{E}$[*Y*|*A*_1_, *A*_2_, *L*_2_ = *l*_2_]*p*(*l*_2_), where data in “Risk” and “*p*(*L*_2_)” columns in each *L*_2_ = *l*_2_ (0 or 1) row are used for E[*Y*|*A*_1_, *A*_2_, *L*_2_ = *l*_2_] and *p*(*l*_2_), respectively.

^d^Calculate $\sum_{l_{2}}\text{E}$[*Y*|*A*_1_, *A*_2_, *L*_2_ = *l*_2_]*p*(*l*_2_|*A*_1_) as above, except for using probabilities in “*p*(*L*_2_|*A*_2_)” instead of “*p*(*L*_2_)” for the corresponding *L*_2_ and *A*_2_ values.

Instead of using *P*(*L*_2_ = *l*_2_) in the standardization formula, the “g-formula” in Table 3 averages the stratified risks E[*Y*|*A*_1_, *L*_2_ = *l*_2_, *A*_2_] using the weights *P*(*L*_2_ = *l*_2_|*A*_1_):

$$\sum_{l_{2}}\text{E}[Y|A_{1}=a_{1},L_{2}=l_{2},A_{2}=a_{2}]P(L_{2}=l_{2}|A_{1}=a_{1}).$$

Unlike the previous two naïve estimates, we can see that these values are equal to E[$Y^{a_{1},a_{2}}$] in Table 2. As elaborated in the next subsection, the g-formula is one expression of E[$Y^{a_{1},a_{2}}$] in terms of observed distribution under the condition that is different from unconditional/conditional exchangeability.

#### Conditions for identification of the effects

Instead of conditional exchangeability E[$Y^{a_{1},a_{2}}$|*A*_1_, *L*_2_, *A*_2_] = E[$Y^{a_{1},a_{2}}$|*L*_2_] for joint exposure, we can easily check the following conditions,

$$\text{E}[Y^{a_{1},a_{2}}|A_{1}=1]=\text{E}[Y^{a_{1},a_{2}}|A_{1}=0]\text{ and}$$ (C1)

$$\begin{array}{rl} & \text{E}[Y^{a_{1},a_{2}}|A_{1}=a_{1},L_{2},A_{2}=1]\\ & \;=\text{E}[Y^{a_{1},a_{2}}|A_{1}=a_{1},L_{2},A_{2}=0], \end{array}$$ (C2)

for all *a*_1_ and *a*_2_, from upper four rows vs lower four rows (for (C1)) and every 2 rows within the same stratum of (*A*_1_, *L*_2_) (for (C2)) in Table 2. These conditions are collectively called the *sequential exchangeability* for (*A*_1_, *A*_2_),^6^^,^^36^^,^^51^ which are typically easier to hold than joint conditional exchangeability but are neither necessary nor sufficient condition for joint conditional exchangeability (see Appendix A for more technical notes on the conditions). The covariates that satisfy (C2) through their stratification (ie, *L*_2_ here) are called *time-varying confounders*. In fact, slightly strong condition (C2′) E[$Y^{a_{1},a_{2}}$|*A*_1_, *L*_2_, *A*_2_ = 1] = E[$Y^{a_{1},a_{2}}$|*A*_1_, *L*_2_, *A*_2_ = 0] (which requires conditional independence in all *A*_1_ supports instead of only in *A*_1_ = *a*_1_ compatible with intervention on $Y^{a_{1},a_{2}}$) also holds in our example, while this is not required for the g-formula to be equal to E[$Y^{a_{1},a_{2}}$]. The g-formula equals E[$Y^{a_{1},a_{2}}$] if sequential exchangeability (C1) and (C2) holds.

**Figure 1.**
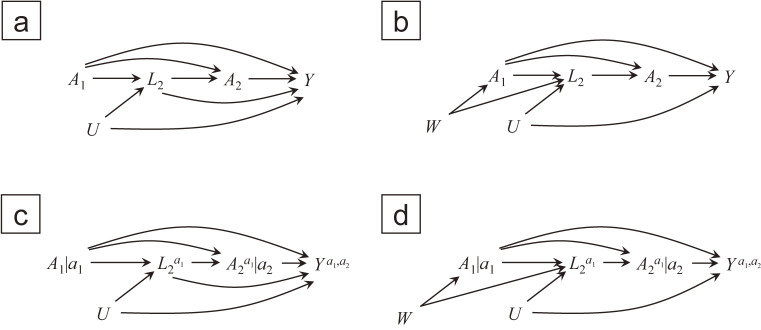
Causal DAGs and SWIGs compatible with example data, where *U* and *W* are unobserved variables: (a) causal DAG without *W*, in which *A*_1_–*L*_2_, *A*_1_–*Y*, and *A*_2_–*Y* are (conditionally) unconfounded given observed data; (b) causal DAG with *W*, in which *A*_1_–*Y* and *A*_2_–*Y* are (conditionally) unconfounded but *A*_1_–*L*_2_ is confounded given observed data; (c) a “template” under intervention (*a*_1_, *a*_2_) of SWIG that corresponds to causal DAG (a); (d) a “template” under intervention (*a*_1_, *a*_2_) of SWIG that corresponds to causal DAG (b).

It is helpful to depict the conditions in causal diagrams, namely, causal directed acyclic graphs (DAGs)^2^^,^^29^ and single-world intervention graphs (SWIGs)^31^^,^^32^; we would like readers unfamiliar with these graphical terminology and rules (eg, opening/blocking paths, d-separation, the backdoor criterion) to refer to introductory articles^30^^,^^32^^,^^35^ or book chapters^6^^,^^34^ on the topic. Informally, variables are *d-separated* if they are not connected with each other or connected only through paths on which at least one unadjusted “colliders” or adjusted “non-colliders” exist. If a supposed exposure is d-separated from a supposed outcome by adjusting for non-descendant variables of the exposure (in an original graph) after deletion of arrows emanating from the exposure, then we would say the *backdoor criterion* is satisfied. Figure 1, which is adopted from Part 3 of *Causal Inference: What If*,^6^ represents the causal diagrams that imply (C1) and (C2). Note that the typical strategy for causal inference in practice starts by drawing a causal DAG (eg, Figure 1(a)) or a SWIG (eg, Figure 1(c)) assumed for the data-generating process. Then, (conditional) independences between potential and observed variables, such as (C1) and (C2), are deduced from the graph. Here, we go backward; we start with counterfactual data (Table 2) in which (C1) and (C2) hold and proceed to causal DAGs/SWIGs that are compatible with those conditions.

In Figure 1(a), there is no non-descendant variable set that blocks all backdoor paths from collective nodes (*A*_1_, *A*_2_) to *Y* (ie, satisfies the backdoor criterion). On the contrary, the backdoor paths to *Y* from *A*_1_ and *A*_2_ are separately blocked by distinct sets of variables: empty set for *A*_1_ and (*A*_1_, *L*_2_) for *A*_2_. The arguments can be more directly depicted using potential variables in Figure 1(c), which is a “template” of the SWIG representing each intervention (*a*_1_, *a*_2_) on (*A*_1_, *A*_2_).^32^ For example, *A*_1_ is d-separated from any variables, and $A_{2}^{a_{1}}$ is d-separated from $Y^{a_{1},a_{2}}$ given $L_{2}^{a_{1}}$. After additionally conditioning on *A*_1_ = *a*_1_ (which is automatically done in the “template”), $A_{2}^{a_{1}}=A_{2}$ (by consistency) is still d-separated from $Y^{a_{1},a_{2}}$ given $L_{2}^{a_{1}}=L_{2}$ (by consistency) and *A*_1_ = *a*_1_; thus, (C1) and (C2) are satisfied in this SWIG. The same arguments can be applied to Figure 1(b) and (d), where *A*_1_–*L*_2_ is confounded (ie, connected by a backdoor path) by unobserved *W*. In other words, there are settings where joint effects of (*A*_1_, *A*_2_) on *Y* can be identified (via sequential exchangeability) even if the effects of *A*_1_ on *L*_2_ are not identifiable (by the unobservable). More implication obtained from Figure 1 is detailed in Appendix B. The remainder of the paper does not require the reference to causal diagrams.

### Different view of the g-formula: inverse probability weighting

We have seen that under the sequential exchangeability (C1) and (C2), the g-formula is equivalent to the averages of potential outcome. If baseline confounders *L*_1_ exist, the g-formula is

$$\begin{array}{rl} & \text{E}[\text{E}[\text{E}[Y|L_{1},A_{1}=a_{1},L_{2},A_{2}=a_{2}]|A_{1}=a_{1},L_{1}]]\\ & \;=\sum_{l_{1}}\sum_{l_{2}}\text{E}[Y|L_{1}=l_{1},A_{1}=a_{1},L_{2}=l_{2},A_{2}=a_{2}]\\ & \;\times P(L_{2}=l_{2}|L_{1}=l_{1},A_{1}=a_{1})P(L_{1}=l_{1}), \end{array}$$ (1)

which is equivalent to E[$Y^{a_{1},a_{2}}$] if (C1) and (C2) hold by additionally conditioning on *L*_1_. The left-hand side of equation (1) is a representation of the iterative conditional expectation of the g-formula.

The alternative expression of E[$Y^{a_{1},a_{2}}$] under (C1) and (C2) is *inverse probability weighting*^6^^,^^42^^,^^51^^,^^64^:

$$\text{E}\left[\frac{I(A_{1}=a_{1},A_{2}=a_{2})}{p(A_{1}|L_{1})p(A_{2}|L_{1},A_{1},L_{2})}Y\right],$$ (2)

where *I*(*A*_1_*_i_* = *a*_1_, *A*_2_*_i_* = *a*_2_) is an indicator function that takes 1 if individual *i* has joint exposure level (*a*_1_, *a*_2_) and 0 otherwise, *p*(*a*_1_|*l*_1_) = *P*(*A*_1_ = *a*_1_|*L*_1_ = *l*_1_) is a conditional probability function of first exposure having level *a*_1_ and *p*(*a*_2_|*l*_1_, *a*_1_, *l*_2_) = *P*(*A*_2_ = *a*_2_|*L*_1_ = *l*_1_, *A*_1_ = *a*_1_, *L*_2_ = *l*_2_) is a conditional probability function of second exposure having level *a*_2_ given past exposure and covariates. Accordingly, *p*(*A*_1_*_i_*|*L*_1_*_i_*) and *p*(*A*_2_*_i_*|*L*_1_*_i_*, *A*_1_*_i_*, *L*_2_*_i_*) in formula (2) are functions of individual data.

These two expressions are equivalent forms of E[$Y^{a_{1},a_{2}}$] under sequential exchangeability (C1) and (C2), as well as the time-varying versions of consistency and positivity. Despite the equivalence of these identification formulas, the estimator that plugs each estimate into (1) is called a g-formula estimator and that based on (2) is an inverse probability weighted estimator. The arguments can be extended to “dynamic regimes” with stronger conditions (Appendix B).^51^^,^^64^

Now, let us obtain inverse probability weighted estimates from Table 2. First, we garner the probability of *actually received* exposure given past exposure and covariates separately for *A*_1_ and *A*_2_. As *L*_1_ is empty to achieve sequential exchangeability, *p*(*A*_1_*_i_*) and *P*(*A*_2_*_i_*|*A*_1_*_i_*, *L*_2_*_i_*) for each combination of (*A*_1_*_i_*, *L*_2_*_i_*, *A*_2_*_i_*) are provided in Table 4. Next, calculate the “inverse probability weights” 1/{*p*(*A*_1_*_i_*)*p*(*A*_2_*_i_*|*A*_1_*_i_*, *L*_2_*_i_*)} and multiply the numbers of combinations (*A*_1_*_i_*, *L*_2_*_i_*, *A*_2_*_i_*) by the weights. Note that the sum of the weights *I*(*A*_1_ = *a*_1_, *A*_2_ = *a*_2_)/{*p*(*A*_1_)*p*(*A*_2_|*A*_1_, *L*_2_)} for each (*a*_1_, *a*_2_) equals total sample size (ie, *n* = 15,000 in our data). Hence, formula (2) indicates that we only have to estimate the probability of *Y* = 1 for every combination of (*a*_1_, *a*_2_) in these multiplied numbers, or the inverse probability weighted population:

$$\begin{array}{rl} {\text{E}}_{\text{IPW}}[Y|A_{1}=1,A_{2}=1] & =(2,400+4,800)/(3,000+12,000)\\ & =7,200/15,000=0.48, \end{array}$$

$$\begin{array}{rl} {\text{E}}_{\text{IPW}}[Y|A_{1}=1,A_{2}=0] & =(1,800+6,000)/(3,000+12,000)\\ & =7,800/15,000=0.52, \end{array}$$

$$\begin{array}{rl} {\text{E}}_{\text{IPW}}[Y|A_{1}=0,A_{2}=1] & =(7,200+2,100)/(9,000+6,000)\\ & =9,300/15,000=0.62, \end{array}$$

$$\begin{array}{rl} {\text{E}}_{\text{IPW}}[Y|A_{1}=0,A_{2}=0] & =(8,100+1,800)/(9,000+6,000)\\ & =9,900/15,000=0.66. \end{array}$$

**Table 4.** Hypothetical cohort data weighted by inverse probability of exposures

*A*_1_	*L*_2_	*A*_2_	Unweighted number		*p*(*A*_1_)	*p*(*A*_2_|*A*_1_, *L*_2_)	IPW	Number multiplied by IPW	
	
			*N*	*Y* = 1				*N*	*Y* = 1
1	1	1	720	576	0.3	0.8	4.17	3,000	2,400
1	1	0	180	108	0.3	0.2	16.67	3,000	1,800
1	0	1	1,800	720	0.3	0.5	6.67	12,000	4,800
1	0	0	1,800	900	0.3	0.5	6.67	12,000	6,000
0	1	1	5,670	4,536	0.7	0.9	1.59	9,000	7,200
0	1	0	630	567	0.7	0.1	14.29	9,000	8,100
0	0	1	840	294	0.7	0.2	7.14	6,000	2,100
0	0	0	3,360	1,008	0.7	0.8	1.79	6,000	1,800

IPW, inverse probability weight.

### Marginal structural models

We have estimated four distinct E[$Y^{a_{1},a_{2}}$] separately via g-formula (1) or inverse probability weighting (2). No approximation, or *model*, has been used.

Now, carefully look at the true values E[$Y^{a_{1},a_{2}}$] in the last row of Table 2. We can see that E[*Y*^1,0^] − E[*Y*^0,0^] = 0.52 − 0.66 = 0.48 − 0.62 = E[*Y*^1,1^] − E[*Y*^0,1^]; the difference between *a*_1_ = 1 vs *a*_1_ = 0 is −14%, irrespective of the value of *a*_2_. Likewise, review E[*Y*^0,1^] − E[*Y*^0,0^] = 0.62 − 0.66 = 0.48 − 0.52 = E[*Y*^1,1^] − E[*Y*^1,0^] and the causal risk difference of *a*_2_ = 1 vs *a*_2_ = 0 is −4%. We can collectively write the counterfactual expectations as follows: E[$Y^{a_{1},a_{2}}$] = 0.66 − 0.14*a*_1_ − 0.04*a*_2_. More generally, we may describe the relation between E[$Y^{a_{1},a_{2}}$] and (*a*_1_, *a*_2_) as

$$\text{E}[Y^{a_{1},a_{2}}]=\beta_{0}+\beta_{1}a_{1}+\beta_{2}a_{2}.$$ (3)

This is the *correctly specified* marginal structural model; if we have the data in Table 2, the parameters of marginal structural model (3) can be unbiasedly estimated by, for example, the least-squares or maximum-likelihood methods. The marginal structural models are the simplified expressions of E[$Y^{a_{1},a_{2}}$] by restricting the possible values of E[$Y^{a_{1},a_{2}}$].^42^^,^^43^^,^^51^ In equation (3), the left-hand side can take any four values, but the right-hand side expresses them by only three parameters. Model (3) is *marginal* because the expectations are taken with the marginal distributions of $Y^{a_{1},a_{2}}$ unconditional on other observed variables (though the condition is relaxed later) and other potential outcomes $Y^{a_{1}^{\prime },a_{2}^{\prime }}$ other than (*a*_1_, *a*_2_) (thus, we need not consider any cross-world joint distributions under different interventions).^42^^,^^46^ Model (3) is also *structural* because it imposes restrictions on potential outcomes $Y^{a_{1},a_{2}}$ rather than observed distributions.

There are other possibilities for specification of marginal structural models. For example, we can fit the simpler additive model

$$\text{E}[Y^{a_{1},a_{2}}]=\beta_{0}+\beta_{1}(a_{1}+a_{2}),$$ (4)

which has only two parameters assuming that *A*_1_ and *A*_2_ have the same effect (risk difference) on *Y*, or a multiplicative marginal structural model

$$\log\text{E}[Y^{a_{1},a_{2}}]=\beta_{0}+\beta_{1}a_{1}+\beta_{2}a_{2},$$ (5)

where exp(β_1_) and exp(β_2_) represent the (common) risk ratios E[$Y^{1,a_{2}}$]/E[$Y^{0,a_{2}}$] (*a*_2_ = 0, 1) and E[$Y^{a_{1},1}$]/E[$Y^{a_{1},0}$] (*a*_1_ = 0, 1), respectively. However, these are *incorrectly specified* or *misspecified* marginal structural models because any parameter values (β_0_, β_1_) or (β_0_, β_1_, β_2_) in the right-hand sides of (4) and (5) cannot exactly express the left-hand sides. A marginal structural model is correctly specified in multiplicative scale by making it *saturated* by, for example, including an interaction term of *a*_1_ and *a*_2_:

$$\log\text{E}[Y^{a_{1},a_{2}}]=\beta_{0}+\beta_{1}a_{1}+\beta_{2}a_{2}+\beta_{3}a_{1}a_{2}.$$ (6)

We estimate these marginal structural models through inverse probability weighting from observed data in Table 3, where sequential exchangeability (C1) and (C2) holds. Of course, models (4) and (5) are misspecified and necessarily result in biased estimates of E[$Y^{a_{1},a_{2}}$]. Nevertheless, the estimates of misspecified marginal structural models may well approximate the true E[$Y^{a_{1},a_{2}}$] unless the model forms differ significantly from the true relationship between E[$Y^{a_{1},a_{2}}$] and (*a*_1_, *a*_2_). A typical estimation process is as follows: 1) calculate the inverse probability weight, 1/{*p*(*A*_1_*_i_*)*p*(*A*_2_*_i_*|*A*_1_*_i_*, *L*_2_*_i_*)}, for each variable pattern (*A*_1_*_i_*, *L*_2_*_i_*, *A*_2_*_i_*) as in Table 4; 2) fit the regression model for E[*Y*|*A*_1_ = *a*_1_, *A*_2_ = *a*_2_] with the same functional form of the marginal structural models; and 3) obtain confidence intervals by the sandwich estimator or bootstrap. The SAS and Stata codes to create a dataset and replicate the results are provided in Appendix C and Supplementary Material, respectively. Table 5 shows the parameter estimates of these models. Expectations E[$Y^{a_{1},a_{2}}$] are also estimated by linear combination of these estimates in the corresponding models; eg, E[*Y*^0,0^] = β_0_ (models 3 and 4) or exp(β_0_) (models 5 and 6), and E[*Y*^1,1^] = β_0_ + β_1_ + β_2_ (model 3), β_0_ + 2β_1_ (model 4), exp(β_0_ + β_1_ + β_2_) (model 5), or exp(β_0_ + β_1_ + β_2_ + β_3_) (model 6).

**Table 5.** Inverse probability weighted estimates of marginal structural models from observed hypothetical cohort data (Table 3)

	MSM (3): Correct		MSM (4): Incorrect		MSM (5): Incorrect		MSM (6): Correct	
			
	Estimate^a^	95% CI^b^	Estimate^a^	95% CI^b^	Estimate^a^	95% CI^b^	Estimate^a^	95% CI^b^
Risk difference or ratio								
*A*_1_ (*a*_1_ = 1 vs 0)	−0.140	−0.160, −0.120	−0.090^c^	−0.104, −0.076	0.781	0.753, 0.810	0.788^d^	0.746, 0.832
*A*_2_ (*a*_2_ = 1 vs 0)	−0.040	−0.060, −0.020	−0.090^c^	−0.104, −0.076	0.932	0.900, 0.965	0.939^e^	0.903, 0.978
*A*_1_*A*_2_	—		—		—		0.983^f^	0.914, 1.057

Potential outcome mean								
E[*Y*^0,0^]	0.660	0.643, 0.677	0.660	0.643, 0.677	0.663	0.645, 0.681	0.660	0.641, 0.680
E[*Y*^0,1^]	0.620	0.605, 0.635	0.570	0.560, 0.580	0.618	0.602, 0.634	0.620	0.604, 0.637
E[*Y*^1,0^]	0.520	0.501, 0.539	0.570	0.560, 0.580	0.518	0.499, 0.537	0.520	0.497, 0.544
E[*Y*^1,1^]	0.480	0.463, 0.497	0.480	0.463, 0.497	0.483	0.466, 0.499	0.480	0.461, 0.500

CI, confidence interval; MSM, marginal structural model.

^a^Risk differences β (MSMs (3) and (4)) or risk ratios exp(β) (MSMs (5) and (6)) in the upper part.

^b^Using sandwich estimator.

^c^Common risk difference for *A*_1_ and *A*_2_.

^d^Risk ratio for *A*_1_ when controlling *A*_2_ at 0: E[*Y*^1,0^]/E[*Y*^0,0^].

^e^Risk ratio for *A*_2_ when controlling *A*_1_ at 0: E[*Y*^0,1^]/E[*Y*^0,0^].

^f^Interaction between *A*_1_ and *A*_2_ in risk ratio scale: (E[*Y*^1,1^]E[*Y*^0,0^])/(E[*Y*^1,0^]E[*Y*^0,1^]).

Why do we need to model E[$Y^{a_{1},a_{2}}$] by taking the risk to cause bias? Consider exposures can change at an additional one time point. Without models, we need to estimate 2^3^ = 8 (double of our case) distinct E[$Y^{a_{1},a_{2},a_{3}}$]. If we have six time points, the task requires 64 estimates from the limited amount of data. Furthermore, if we have continuous exposure, we have to rely on the dose-response curves irrespective of the number of exposure time points. Given we always have a limited amount of data, our estimation tasks must rely on the dimension reduction of parameter space by imposing restriction on the possible values of counterfactual outcome means. In Table 5, despite both models (3) and (6) being correctly specified and unbiasedly estimated, the estimates of E[$Y^{a_{1},a_{2}}$] from model (3) (3 parameters) have slightly narrower confidence intervals than those from model (6) (four parameters). The efficiency gain owing to dimension reduction will be modest as the number of time points increases.

Note that models (3)–(6) do not require covariate information, though can incorporate baseline confounders *L*_1_ for examining effect modifications by certain variables in specific scales (eg, risk difference or ratio).^6^^,^^68^ The convenient choice that is commonly seen in practice may be the simplest model assuming a common exposure effect across time and baseline confounder strata:

$$\text{E}[Y^{a_{1},a_{2}}|L_{1}=l_{1}]=\beta_{0}+\beta_{1}(a_{1}+a_{2})+\beta_{2}{}^{\text{T}}l_{1},$$

which imposes more restriction than the marginal structural model (4), which is agnostic about (ie, does not assume) no-effect modification by *L*_1_. To assess effect modification by baseline confounders, the model can be modified as

$$\text{E}[Y^{a_{1},a_{2}}|L_{1}=l_{1}]=\beta_{0}+\beta_{1}(a_{1}+a_{2})+\beta_{2}{}^{\text{T}}l_{1}+\beta_{3}{}^{\text{T}}(a_{1}+a_{2})l_{1},$$

though this is still generally stricter than model (4) because the effect of exposure is restricted to be linearly modified by *L*_1_.

### Dealing with high-dimensional covariates

In our example, we have no baseline confounder and only one time-varying binary confounder variable *L*_2_, as well as two binary exposures *A*_1_ and *A*_2_. As a result, we can estimate all conditional expectations and conditional probabilities in g-formula (1) and inverse probability weighting (2) from the direct calculation of the mean/proportion in each stratum; in other words, we used saturated regression and exposure probability models. In practice, however, we have many variables in *L*_1_ or *L*_2_, or both, some of which may follow continuous or multinomial distributions. In such cases, we must rely on models for observed distribution of (*L*_1_, *A*_1_, *L*_2_, *A*_2_, *Y*).^4^^,^^20^^,^^69^^,^^70^

For example, g-formula (1) can be estimated by fitting the following outcome and covariate regression models:

$$\begin{array}{rl} & \text{E}[Y|L_{1}=l_{1},A_{1}=a_{1},L_{2}=l_{2},A_{2}=a_{2}]\\ & \;=\gamma_{0}+\gamma_{1}{}^{\text{T}}l_{1}+\gamma_{2}a_{1}+\gamma_{3}{}^{\text{T}}l_{2}+\gamma_{4}a_{2} \end{array}$$

$$\begin{array}{rl} & \text{E}[L_{2k}|L_{1}=l_{1},A_{1}=a_{1},L_{20}=l_{20},\ldots ,L_{2,k-1}=l_{2,k-1}]\\ & \;=\left\{ \begin{array}{c} \delta_{k0}+\delta_{k1}^{\text{T}}l_{1}+\delta_{k2}a_{1}\;\text{(for }k=1\text{)}\\ \delta_{k0}+\delta_{k1}^{\text{T}}l_{1}+\delta_{k2}a_{1}+\sum_{j=1}^{k-1}\delta_{k3j}l_{2j}\;\text{(for }k=2,\ldots ,K\text{)} \end{array}\right. \end{array}$$

where *L*_2_*_k_* is a *k*th variable in arbitrarily ordered *L*_2_ = (*L*_21_,…, *L*_2_*_K_*)^T^ with a constant *L*_20_. Note that in general, we must conduct numerical approximation of conditional distribution of *L*_2_*_k_* by simulating the Monte–Carlo samples from the model fit, which has the conditional means following the above regression models (the parametric g-formula estimator).^38^^,^^47^ Alternatively, we could iteratively model the left-hand side of g-formula (1) from inside to outside of expectations by fitting the outcome regression models for the predictions from previous model fit (equivalent to the Q-learning estimator).^24^^,^^71^

Inverse probability weighting formula (2) can also be estimated by, for example, logistic models for exposure probabilities:

$$logit\;P(A_{1}=1|L_{1}=l_{1})=\alpha_{10}+\alpha_{11}{}^{\text{T}}l_{1},$$

$$\begin{array}{rl} & logit\;P(A_{2}=1|L_{1}=l_{1},A_{1}=a_{1},L_{2}=l_{2})\\ & \;=\alpha_{20}+\alpha_{21}{}^{\text{T}}l_{1}+\alpha_{22}a_{1}+\alpha_{23}{}^{\text{T}}l_{2}. \end{array}$$

We then calculate the weighted mean using 1/{*p*(*A*_1_*_i_*|*L*_1_*_i_*)*p*(*A*_2_*_i_*|*L*_1_*_i_*, *A*_1_*_i_*, *L*_2_*_i_*)} from predicted values from these models.

Note that both of these approaches do not impose any restriction on the values of E[$Y^{a_{1},a_{2}}$]; we could use regression or exposure probability models without specifying marginal structural models and *vice versa* (recall the calculation of Table 5). Marginal structural models are causal assumptions about the relationship between E[$Y^{a_{1},a_{2}}$] and hypothetical intervention (*a*_1_, *a*_2_); on the contrary, regression and exposure probability models are approximations of certain aspects of the observed distribution of (*L*_1_, *A*_1_, *L*_2_, *A*_2_, *Y*). In practice, however, we should rely on both marginal structural models and exposure probability models when using inverse probability weighting for estimating the effects of exposure with a moderate number of time points.^44^^–^^50^

Table 6 shows the estimates of marginal structural models (3)–(6) using the fit of a misspecified exposure probability model: logit *P*(*A*_2_ = 1|*A*_1_ = *a*_1_, *L*_2_ = *l*_2_) = α_0_ + α_1_*a*_1_ + α_2_*l*_2_. As expected, all estimates of E[$Y^{a_{1},a_{2}}$] are biased from Table 2 owing to the exposure probability model misspecification. Moreover, even for correctly specified marginal structural models (3) and (6), these estimates diverge from each other when using an exposure probability model to estimate inverse probability weights. Similar to the dimension reduction via marginal structural models, we would expect a greater efficiency gain (ie, variance reduction) in inverse probability weighting estimators when high-dimensional confounders must be conditioned on to achieve sequential exchangeability.

**Table 6.** Inverse probability weighted estimates of marginal structural models using a misspecified exposure probability model

	MSM (3): Correct		MSM (4): Incorrect		MSM (5): Incorrect		MSM (6): Correct	
			
	Estimate^a^	95% CI^b^	Estimate^a^	95% CI^b^	Estimate^a^	95% CI^b^	Estimate^a^	95% CI^b^
Risk difference or ratio								
*A*_1_ (*a*_1_ = 1 vs 0)	−0.119	−0.145, −0.092	−0.081^c^	−0.095, −0.068	0.813	0.774, 0.855	0.886^d^	0.819, 0.958
*A*_2_ (*a*_2_ = 1 vs 0)	−0.045	−0.073, −0.017	−0.081^c^	−0.095, −0.068	0.924	0.880, 0.969	1.022^e^	0.983, 1.063
*A*_1_*A*_2_	—		—		—		0.822^f^	0.749, 0.902

Potential outcome mean								
E[*Y*^0,0^]	0.655	0.635, 0.674	0.649	0.628, 0.671	0.658	0.638, 0.679	0.625	0.606, 0.644
E[*Y*^0,1^]	0.610	0.593, 0.628	0.568	0.552, 0.584	0.608	0.590, 0.626	0.639	0.624, 0.654
E[*Y*^1,0^]	0.536	0.503, 0.570	0.568	0.552, 0.584	0.535	0.502, 0.569	0.554	0.515, 0.595
E[*Y*^1,1^]	0.491	0.472, 0.511	0.487	0.466, 0.507	0.494	0.475, 0.513	0.465	0.445, 0.486

CI, confidence interval; MSM, marginal structural model.

^a^Risk differences β (MSMs (3) and (4)) or risk ratios exp(β) (MSMs (5) and (6)) in the upper part.

^b^Using sandwich estimator.

^c^Common risk difference for *A*_1_ and *A*_2_.

^d^Risk ratio for *A*_1_ when controlling *A*_2_ at 0: E[*Y*^1,0^]/E[*Y*^0,0^].

^e^Risk ratio for *A*_2_ when controlling *A*_1_ at 0: E[*Y*^0,1^]/E[*Y*^0,0^].

^f^Interaction between *A*_1_ and *A*_2_ in risk ratio scale: (E[*Y*^1,1^]E[*Y*^0,0^])/(E[*Y*^1,0^]E[*Y*^0,1^]).

### Summary of pitfalls and tips

Our hypothetical dataset explicitly shows estimands (ie, E[$Y^{a_{1},a_{2}}$]) and minimally possesses the counterfactual conditions (ie, sequential exchangeability) to estimate counterfactual means under joint intervention on time-varying exposure (*A*_1_, *A*_2_). We hitherto illustrate the tips (Box) for formal understanding of marginal structural modeling and its estimation through inverse probability weighting (pitfall 1), as well as the required causal assumptions on unobservable data. Models are used to account for the “curse of dimensionality.” On one hand, marginal structural models reduce the dimension of counterfactual outcome means under a huge number of the combinations of time-varying exposures. On the other hand, exposure probability models must be adopted in practice to account for the large numbers of baseline and time-varying confounders, which usually do not have implications on marginal structural modeling (pitfalls 2 and 4). We also show the biases based on the misspecification of exposure probability models and misspecification of marginal structural models separately (pitfall 3). Note that while inverse probability weighting and the g-formula are applicable to estimate marginal counterfactual means (ie, saturated marginal structural models), only the former can estimate general, unsaturated marginal structural models (pitfall 5). Although running into these pitfalls may not necessarily lead to large biases in practical analysis, failure to recognize these subtleties would advocate unprincipled and suboptimal strategies for causal inference.

**Box.** Key messages for clear understanding of marginal structural modeling

•	Marginal structural models (MSMs) should be distinguished from inverse probability weighting
•	MSM shows prespecified assumptions on causal estimands, while an exposure probability model is an imposed restriction on observed distribution
•	As MSM and exposure probability model are used for different purposes, misspecification of these models would lead to biases in different ways
•	Model specifications of MSMs and exposure probability models raise different challenges in real data analysis
•	G-formula, which shares identifiability assumptions with inverse probability weighting, can be used to fit MSMs only when the models are saturated

We would conclude this section with additional emphases of two pitfalls. First, variable selection and model specification are generally different tasks in modeling for causal inference. By inverse probability weighting, exposure probability models should select confounders, stratification of which is sufficient to achieve sequential exchangeability. In our example, all analyses with or without an exposure probability model include all confounder(s), *L*_2_. Even if the models include all confounders, however, they may be misspecified as in the analysis in Table 6. The same is true for regression models for the g-formula. On the contrary, it is unnecessary for marginal structural models to include confounders; only covariates (need not to be confounders but should be conditioned in propensity score^19^) that may modify the exposure effect of interest may be included in marginal structural models.^6^^,^^68^

Second, *doubly robust estimators* can alleviate the bias from misspecification of regression and exposure probability models,^22^^–^^28^ but not the bias owing to the misspecification of marginal structural models nor other causal models (that are not introduced in this paper). For example, Table 6 provides the biased estimates using a misspecified exposure probability model for correct/incorrect marginal structural models. Among them, bias in the estimates of correct marginal structural models (3) and (6) would be mitigated by doubly robust methods, by including outcome regression models via the iterative model-fitting algorithm of Bang and Robins,^24^ while the fitting of incorrect marginal structural models (4) and (5) must result in biased estimates. Hence, even with doubly robust methods, the careful consideration of marginal structural models is needed, especially for long-term follow-up study with many time points at which exposure can change. Marginal structural models for dynamic regimes may also have to depend on strong modeling assumptions,^51^^,^^64^^,^^72^^–^^74^ even when exposure is binary and change at several time points.

## FUTURE DIRECTIONS

There is a relevant method other than the g-formula and inverse probability weighting that requires essentially the same assumptions to estimate causal effects of time-varying exposures: g-estimation.^15^^,^^18^^,^^38^^–^^41^^,^^51^^,^^67^ Like the relation of marginal structural modeling and inverse probability weighting, g-estimation is a method to estimate the parameters of *structural nested models*. Structural nested models and g-estimation indeed have attractive statistical properties (eg, robustness, efficiency, and flexible parameterization), which successfully work within Robins’ causal “interventionism” framework with minimal conditions.^31^^,^^41^^,^^46^^,^^63^^,^^75^ Despite its theoretical superiority, g-estimation has been underused in epidemiologic literature probably because of the complexity of background theory and interpretability of the parameters.^75^ However, structural nested models are especially useful for dynamic regimes of time-varying exposures by modeling the effect modification by time-varying covariates,^38^^,^^41^^,^^51^ which cannot generally be included in marginal structural models.^46^^,^^68^

Besides the conceptual pitfalls considered in this paper, there are important pitfalls regarding specification and estimation of marginal structural models, which will often lead to mistakes in practice:

• One should always use the independence working correlations in marginal structural models of repeated-measures outcomes.^47^^,^^76^^,^^77^
• If “stabilized” weights include covariates in the numerator weights,^43^ they should be conditioned in the marginal structural models.^50^
• “Stabilization” of the weights is not always acceptable (eg, dynamic-regime marginal structural models^72^^–^^74^).
• It is always important to check the fits of exposure probability models (eg, checking calibration or model-diagnostic measures^78^ and weight distributions^50^) and marginal structural models (eg, comparing the estimating equation-based quasi-likelihood information criterion with that for less restricted models^79^ or testing equivalence between asymptotic values of parameter estimates obtained through different weighting options^80^).

There are other practical concerns in real data analysis. For example, many follow-up studies compare time-to-event outcomes, which complicate the modeling and estimation process for the effects of time-varying exposure. In these settings, time-dependent Cox models or the risk-set switching Kaplan–Meier estimators would need unrealistic assumptions to yield causally interpretable estimates.^43^^,^^81^ In addition, censoring of the events must be taken with care by, for example, constructing the inverse probability weights to prevent attrition bias.^44^^,^^45^^,^^51^ Note that the idea of inverse probability of censoring weights appears in diverse causal inference fields; eg, adjustment for treatment discontinuation in clinical trials,^82^^,^^83^ estimation of the effects of dynamic regimes,^72^ and the effects of the treatment duration on survival.^84^

## Figures and Tables

**Appendix Figure 1.**
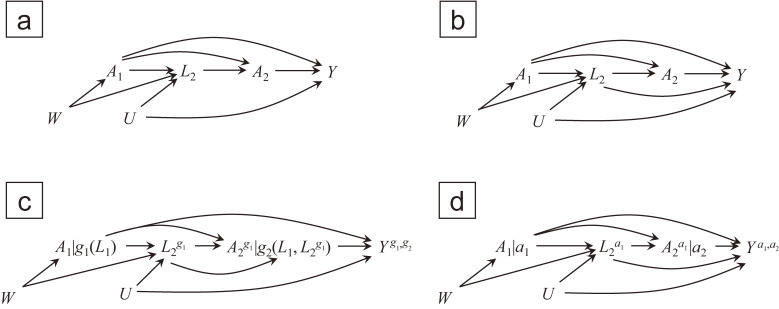
Causal DAGs and SWIGs for dynamic regimes and without identifiability conditions: (a) causal DAG identical to Figure 1(b); (b) causal DAG with the arrow from *L*_2_ on *Y*, in which *L*_2_ is affected by *A*_1_, and the *A*_1_–*L*_2_ association is confounded by unobserved *W*; (c) a “template” under intervention *g* = (*g*_1_, *g*_2_) = (*g*_1_(*L*_1_), *g*_2_(*L*_1_, *A*_1_, *L*_2_)) of SWIG that corresponds to causal DAG (a); (d) a “template” under intervention (*a*_1_, *a*_2_) of SWIG that corresponds to causal DAG (b).

## APPENDIX A. EXCHANGEABILITY CONDITIONS FOR IDENTIFYING THE EFFECTS OF TIME-VARYING EXPOSURES

As shown in Figure 1, sequential exchangeability (C1) and (C2) is more likely in practice than conditional exchangeability E[$Y^{a_{1},a_{2}}$|*A*_1_, *L*_2_, *A*_2_] = E[$Y^{a_{1},a_{2}}$|*L*_2_] for joint exposure (*A*_1_, *A*_2_); conditional exchangeability for joint exposure is not a necessary condition for sequential exchangeability, which would be intuitively understandable to many readers. Mathematically, however, conditional exchangeability for joint exposure itself is not a sufficient condition for sequential exchangeability, either. Nevertheless, these conditions are closely related with each other in other realistic situations, as shown subsequently.

First note that conditional exchangeability always implies (C2), which is rewritten as E[$Y^{a_{1},a_{2}}$|*A*_1_ = *a*_1_, *L*_2_, *A*_2_] = E[$Y^{a_{1},a_{2}}$|*A*_1_ = *a*_1_, *L*_2_]. The right-hand side is E[$Y^{a_{1},a_{2}}$|*A*_1_ = *a*_1_, *L*_2_] = $\sum_{a_{2}^{\prime }}\text{E}$[$Y^{a_{1},a_{2}}$|*A*_1_ = *a*_1_, *L*_2_, *A*_2_ = $a_{2}^{\prime }$]*P*(*A*_2_ = $a_{2}^{\prime }$|*A*_1_ = *a*_1_, *L*_2_) = E[$Y^{a_{1},a_{2}}$|*L*_2_]$\sum_{a_{2}^{\prime }}P$(*A*_2_ = $a_{2}^{\prime }$|*A*_1_ = *a*_1_, *L*_2_) = E[$Y^{a_{1},a_{2}}$|*L*_2_] = E[$Y^{a_{1},a_{2}}$|*A*_1_ = *a*_1_, *L*_2_, *A*_2_] (the left-hand side) using E[$Y^{a_{1},a_{2}}$|*A*_1_, *L*_2_, *A*_2_] = E[$Y^{a_{1},a_{2}}$|*L*_2_]. On the other hand, the right-hand side of the equation E[$Y^{a_{1},a_{2}}$|*A*_1_] = E[$Y^{a_{1},a_{2}}$] (an equivalent form of (C1)) is E[$Y^{a_{1},a_{2}}$] = $\sum_{a_{1}^{\prime },l_{2},a_{2}^{\prime }}\text{E}$[$Y^{a_{1},a_{2}}$|*A*_1_ = $a_{1}^{\prime }$, *L*_2_ = *l*_2_, *A*_2_ = $a_{2}^{\prime }$]*P*(*A*_1_ = $a_{1}^{\prime }$, *L*_2_ = *l*_2_, *A*_2_ = $a_{2}^{\prime }$) = $\sum_{a_{1}^{\prime },l_{2},a_{2}^{\prime }}\text{E}$[$Y^{a_{1},a_{2}}$|*A*_1_ = *a*_1_, *L*_2_ = *l*_2_, *A*_2_ = *a*_2_]*P*(*A*_1_ = $a_{1}^{\prime }$, *L*_2_ = *l*_2_, *A*_2_ = $a_{2}^{\prime }$) (by conditional exchangeability) = $\sum_{l_{2}}\text{E}$[$Y^{a_{1},a_{2}}$|*A*_1_ = *a*_1_, *L*_2_ = *l*_2_, *A*_2_ = *a*_2_]*P*(*L*_2_ = *l*_2_) = $\sum_{l_{2}}\text{E}$[$Y^{a_{1},a_{2}}$|*A*_1_ = *a*_1_, *L*_2_ = *l*_2_]*P*(*L*_2_ = *l*_2_) (using (C2) implied by conditional exchangeability) but cannot further reduce to E[$Y^{a_{1},a_{2}}$|*A*_1_]. However, we can see that 1) if *A*_1_ is independent of $Y^{a_{1},a_{2}}$ (as in Figure 1) or 2) if *P*(*L*_2_ = *l*_2_) = *P*(*L*_2_ = *l*_2_|*A*_1_), that is, *A*_1_ is independent of *L*_2_ in observed data, then (C1) is also implied by conditional exchangeability. Moreover, if *A*_1_ is randomized (ie, ($Y^{a_{1},a_{2}}$, $L_{2}^{a_{1}}$) ⊥⊥ *A*_1_ holds, where “⊥⊥” means statistical independence), then the previous independence condition *P*(*L*_2_ = *l*_2_) = *P*(*L*_2_ = *l*_2_|*A*_1_) is equivalent to (sharp) null effect of *A*_1_ on *L*_2_ by the “g-null” theorem under the faithfulness assumption.^36^^,^^37^ In this case of no-effect of randomized *A*_1_ on time-varying confounders *L*_2_, (C2) implies E[$Y^{a_{1},a_{2}}$|*L*_2_] = E[$Y^{a_{1},a_{2}}$|*A*_1_, *L*_2_] (by randomization) = E[$Y^{a_{1},a_{2}}$|*A*_1_, *L*_2_, *A*_2_]; hence, sequential exchangeability also implies conditional exchangeability for joint exposure.

## APPENDIX B. INDEPENDENCY ASSUMPTIONS ENCODED IN CAUSAL DIAGRAMS AND IDENTIFIABILITY OF GENERAL INTERVENTION REGIMES

Sequential exchangeability (C1) and (C2) is insufficient for identification of the effects of more general exposure interventions (also known as *dynamic regimes* or *strategies*) that may depend on (time-varying) covariates, say, (*L*_1_, *L*_2_). That identification is built on the identification of the distribution *f*($Y^{a_{1},a_{2}}$, $L_{2}^{a_{1}}$), or generally, *f*(*Y^g^*, $L_{2}^{g}$) with the intervention *g* = (*g*_1_(*L*_1_), *g*_2_(*L*_1_, *A*_1_, *L*_2_)), where *g_k_*(·) corresponds to the intervention on *A_k_* possibly depending on past *A* and *L* values (rather than a prespecified value like *a_k_*). Hence, we need more assumptions to identify the effects of a dynamic regime *g*, one of the sufficient conditions is

$$(Y^{a_{1},a_{2}},L_{2}^{a_{1}})⊥\;\;\;⊥A_{1}\text{ and }Y^{a_{1},a_{2}}⊥\;\;\;⊥A_{2}|A_{1}=a_{1},L_{2},$$ (C3)

where *Z*_1_ ⊥⊥ *Z*_2_|*Z*_3_ refers to statistical independence between *Z*_1_ and *Z*_2_ conditional on *Z*_3_.^51^ However, our example is also compatible both with (C3) and the settings with E[$L_{2}^{a_{1}}$|*A*_1_] ≠ E[$L_{2}^{a_{1}}$], in other words, agnostic about condition (C3). Thus, data in Table 2 themselves are not sufficient for the validity of the g-formula for effects of a general regime *g*.

On the contrary, causal diagrams would indicate whether condition (C3) holds and the assumptions encoded in the diagrams allow *f*(*Y^g^*, $L_{2}^{g}$) to be identifiable. From the SWIG of Figure 1(c), we can read the independences ($Y^{a_{1},a_{2}}$, $L_{2}^{a_{1}}$) ⊥⊥ *A*_1_ and $Y^{a_{1},a_{2}}$ ⊥⊥ $A_{2}^{a_{1}}$|*A*_1_ = *a*_1_, *L*_1_, which imply (C3) by consistency under conditioning on *A*_1_ = *a*_1_ (ie, the “world” represented by the SWIG) in the second condition. Thus, the corresponding causal DAG of Figure 1(a) allows us to identify E[*Y^g^*]. However, we cannot deduce (C3) from Figure 1(d) owing to a d-connected path between $L_{2}^{a_{1}}$ and *A*_1_; hence, under the corresponding causal DAG of Figure 1(b), E[*Y^g^*] for a general regimes *g* cannot be identified even though E[$Y^{a_{1},a_{2}}$] for non-dynamic exposure intervention (*a*_1_, *a*_2_) is identified as illustrated in the main text. That is, Figure 1 is one of the examples of causal diagrams that are compatible with our example data, where the stronger causal assumptions are implicitly imposed on. As we have documented earlier,^35^ causal diagrams (when tied with underlying causal models) often represent the “finer” description of causal assumptions than counterfactual notation.

The difficulty in identification of E[*Y^g^*] with *g* = (*g*_1_, *g*_2_) = (*g*_1_(*L*_1_), *g*_2_(*L*_1_, *A*_1_, *L*_2_)) is directly depicted in Appendix Figure 1(a) and (c), where *L*_1_ is suppressed for simplicity but it can affect any variable in the graphs. A causal DAG of Appendix Figure 1(a) is the same as Figure 1(b), while the corresponding SWIGs are different. The structural distinction is the presence (Appendix Figure 1(c)) or absence (Figure 1(d)) of an arrow from *L*_2_ to hypothetical intervention (*g*_2_ or *a*_2_, according to the dependence of intervention on covariates), respectively. We can easily see that dependence between $Y^{g}=Y^{g_{1},g_{2}}$ and *A*_1_ is either with or without conditioning on $L_{2}^{g_{1}}=L_{2}$ in Appendix Figure 1(c), which suggests that E[*Y^g^*] is not identifiable without referring to condition (C3).

Finally, we show a slightly modified causal DAG of Figure 1(b) in Appendix Figure 1(b), in which *L*_2_ that is affected by *A*_1_ also affects *Y*. The corresponding SWIG, Appendix Figure 1(d), reveals that $Y^{a_{1},a_{2}}$ is d-connected either with or without conditioning on $L_{2}^{a_{1}}=L_{2}$; hence, the effects of dynamic regimes *g* and non-dynamic exposure intervention (*a*_1_, *a*_2_) is unidentifiable if the association between exposure and its effect lying on a path to the outcome is confounded by unobservables. Of course, our example data in Table 2 is incompatible with Appendix Figure 1(b) and (d) because of independence between *A* and $Y^{a_{1},a_{2}}$.

## APPENDIX C. SAS CODE FOR HYPOTHETICAL DATA ANALYSIS

* Create a dataset;

data MSM;

  input A1 L2 A2 N N1;

  cumA = A1 + A2;

  do i = 1 to N1; Y = 1; ID + 1; output; end;

  do i = N1 + 1 to N; Y = 0; ID + 1; output; end;

  drop i N N1;

cards;

1  1  1  720  576

1  1  0  180  108

1  0  1  1800   720

1  0  0  1800   900

0  1  1  5670   4536

0  1  0  630  567

0  0  1  840  294

0  0  0  3360   1008

;

* Estimate sequential exposure probabilities;

proc logistic data = MSM desc;

  model A1 = ;

  output out = MSM p = P1;

run;

proc logistic data = MSM desc;

/* Use either of the following two commands */

  model A2 = A1 L2 A1*L2; *Fitting correct exposure probability model for Table 5;

  *model A2 = A1 L2; * Fitting misspecified exposure probability model for Table 6;

  output out = MSM p = P2;

run;

* Calculate inverse probability weights;

data MSM;

  set MSM;

  IPW = (A1/P1 + (1 − A1)/(1 − P1))*(A2/P2 + (1 − A2)/(1 − P2));

run;

* Fit marginal structural model (3): Correct specification;

proc genmod data = MSM;

  class ID;

  model Y = A1 A2 /dist = normal;

  weight IPW;

  repeated sub = ID;

  estimate "E[Y00]" int 1 A1 0 A2 0;

  estimate "E[Y01]" int 1 A1 0 A2 1;

  estimate "E[Y10]" int 1 A1 1 A2 0;

  estimate "E[Y11]" int 1 A1 1 A2 1;

run;

* Fit marginal structural model (4): Misspecification;

proc genmod data = MSM;

  class ID;

  model Y = cumA /dist = normal;

  weight IPW;

  repeated sub = ID;

  estimate "E[Y00]" int 1 cumA 0;

  estimate "E[Y01]" int 1 cumA 1;

  estimate "E[Y10]" int 1 cumA 1;

  estimate "E[Y11]" int 1 cumA 2;

run;

* Fit marginal structural model (5): Misspecification;

proc genmod data = MSM;

  class ID;

  model Y = A1 A2 /dist = Poisson;

  weight IPW;

  repeated sub = ID;

  estimate "A1" A1 1 / exp;

  estimate "A2" A2 1 / exp;

  estimate "E[Y00]" int 1 A1 0 A2 0;

  estimate "E[Y01]" int 1 A1 0 A2 1;

  estimate "E[Y10]" int 1 A1 1 A2 0;

  estimate "E[Y11]" int 1 A1 1 A2 1;

run;

* Fit marginal structural model (6): Correct specification;

proc genmod data = MSM;

  class ID;

  model Y = A1 A2 A1*A2 /dist = Poisson;

  weight IPW;

  repeated sub = ID;

  estimate "A1" A1 1 / exp;

  estimate "A2" A2 1 / exp;

  estimate "A1A2" A1*A2 1 / exp;

  estimate "E[Y00]" int 1 A1 0 A2 0 A1*A2 0;

  estimate "E[Y01]" int 1 A1 0 A2 1 A1*A2 0;

  estimate "E[Y10]" int 1 A1 1 A2 0 A1*A2 0;

  estimate "E[Y11]" int 1 A1 1 A2 1 A1*A2 1;

run;

## APPENDIX D. SUPPLEMENTARY DATA

The following is the supplementary data related to this article:

Supplementary Material: Stata code for hypothetical data analysis
