# Supplementary material for: Understanding Marginal Structural Models for Time-Varying Exposures: Pitfalls and Tips
Source: J Epidemiol. 2020 Sep 5;30(9):377–89. doi: 10.2188/jea.JE20200226 (PMC7429147; doi:10.2188/jea.JE20200226)
Supplement: Supplementary file 1 [file je-30-377-s001.pdf]

Understanding Marginal Structural Models for Time-Varying Exposures: Pitfalls and Tips

Tomohiro Shinozaki and Etsuji Suzuki

Supplementary Material:

Stata code for hypothetical data analysis

```
version 16.1

* Create a dataset
input A1 L2 A2 N N1
1 1 1 720 576
1 1 0 180 108
1 0 1 1800 720
1 0 0 1800 900
0 1 1 5670 4536
0 1 0 630 567
0 0 1 840 294
0 0 0 3360 1008
end
expand N1 + 1, generate(Y)
expand N - N1 if Y == 0
drop N N1
gen cumA = A1 + A2, before(Y)
gsort -A1 -L2 -A2 -Y

* Estimate sequential exposure probabilities
logit A1
predict P1, p

/* Use either of the following two commands */
logit A2 i.A1 i.L2 A1#L2  // Fitting correct exposure probability model for Table 5
*logit A2 i.A1 i.L2      // Fitting misspecified exposure probability model for Table 6
predict P2, p

* Calculate inverse probability weights
gen IPW = (A1/P1 + (1-A1)/(1-P1))*(A2/P2 + (1-A2)/(1-P2))
```

```
* Fit marginal structural model (3): Correct specification
regress Y i.A1 i.A2 [pweight = IPW]
margins A1#A2

* Fit marginal structural model (4): Misspecification
regress Y cumA [pweight = IPW]
margins, at(cumA=(0 1 2))

* Fit marginal structural model (5): Misspecification
poisson Y i.A1 i.A2 [pweight = IPW], irr
margins A1#A2

* Fit marginal structural model (6): Correct specification
poisson Y i.A1 i.A2 A1#A2 [pweight = IPW], irr
margins A1#A2
```
